# Alleviation of *Phytophthora infestans* Mediated Necrotic Stress in the Transgenic Potato (*Solanum tuberosum* L.) with Enhanced Ascorbic acid Accumulation

**DOI:** 10.3390/plants8100365

**Published:** 2019-09-23

**Authors:** Ill-Min Chung, Baskar Venkidasamy, Chandrama Prakash Upadhyaya, Gurusaravanan Packiaraj, Govindasamy Rajakumar, Muthu Thiruvengadam

**Affiliations:** 1 Department of Applied Bioscience, College of Life and Environmental Sciences, Konkuk University, Seoul 05029, Koreamicrolabsraj@gmail.com (G.R.); 2 Department of Biotechnology, Bharathiar University, Coimbatore 641046, Tamil Nadu, India; baskarbt83@gmail.com; 3 Laboratory of Plant Molecular Biology, Department of Biotechnology, Dr. Harisingh Gour Central University, Sagar 470003, Madhya Pradesh, India; 4 Department of Botany, Bharathiar University, Coimbatore 641046, Tamil Nadu, India

**Keywords:** ascorbic acid, *Phytophthora infestans*, reactive oxygen species, *Solanum tuberosum*, transgenic plants

## Abstract

Potato is the most widely cultivated non-cereal crop in the world, and like any other crop, it is susceptible to yield losses because of various factors, including pathogen attacks. Among the various diseases of potato, late blight caused by the oomycete *Phytophthora infestans* is considered as the most devastating disease worldwide. In this study, transgenic potato plants overexpressing the *D-galacturonic acid reductase* (*GalUR*) gene with an enhanced level of cellular L-ascorbate (L-AsA) were challenged with *Phytophthora infestans* to determine the level of stress tolerance induced in those plants. With the onset of pathogen infection, necrotic lesions progressively expanded and became necrotic in the control plants. The transgenic potato lines with enhanced ascorbic acid showed reduced necrotic lesions. Hydrogen peroxide (H_2_O_2_) and malondialdehyde (MDA) levels were relatively lower in transgenic plants compared to the untransformed control (UT) plants. The mRNA expressions of pathogenesis-related (*PR*) genes, such as pathogenesis related 1 (*PR1*) and phenylalanine ammonia-lyase (*PAL*) were slightly higher in *GalUR* overexpressing transgenic lines as compared to the untransformed control plants. Pathogen infection also altered the mRNA expression of genes associated with gibberellic acid (GA) and abscisic acid (ABA) biosynthesis. Furthermore, the increase in various antioxidant enzymes was also observed in the gene expression analysis with the transgenic plants. The complete loss of the pathogen growth and disease occurrence was not observed in our study; however, the findings indicated that an increase in the level of cellular L-ascorbate in the transgenic potato leads to enhanced cellular antioxidants, PR genes and plant defense hormones, such as GA and ABA resulting in the reduction of the disease symptoms caused by the *Phytophthora infestans*.

## 1. Introduction

Potato (*Solanum tuberosum* L.) stands as the world’s fourth largest grown non-cereal crop [1]. Plant development and production of potatos are significantly affected by both abiotic and biotic stresses [2]. In response to pathogen infection, plants trigger defense responses via the production of various reactive oxygen species (ROS), namely hydrogen peroxide (H_2_O_2_), OH^−^, and O_2_^−^ molecules. These ROS act as signals for the induction of a hypersensitive response (HR) mediated cell death and activation of defense associated genes to inhibit pathogen growth and their further spreading [3]. Although these active oxygen species act as a signal in favor of pathogen resistance and other normal physiological processes, their exceedance causes potential damage to biological supermolecules for instance lipids, proteins, and nucleic acids [4]. When the ROS activity exceeds beyond their normal levels, antioxidants, for example, ascorbate, glutathione, α-tocopherol, ß-carotene, and antioxidative enzymes, such as superoxide dismutase (*SOD*), catalases (*CAT*), and peroxidases (*POX*) contribute to the detoxification of ROS activity [5,6]. Among the antioxidants, vitamin C (L-ascorbic acid, AsA) is a significant multifunctional antioxidant compound and an important substrate for the detoxification of reactive oxygen species (ROS) involved in stress tolerance [7,8]. It also acts as a signaling molecule in several physiological processes such as cell division, growth regulation, and senescence [9,10]. It is a cofactor for the enzymes violaxanthin de-epoxidase (catalyst for zeaxanthin production), 1-aminocyclopropane-1-carboxylic acid (*ACC*) oxidase (one of the catalysts in ethylene biosynthesis), and 2-oxoacid-dependent dioxygenases which is involved in abscisic acid (ABA) and gibberellic acid (GA) biosynthesis [11,12,13]. Numerous reports indicate the positive role of AsA and other antioxidants in governing protection to plants against diseases [14].

An exogenous application of AsA, dehydroascorbate, and H_2_O_2_ efficiently alleviated the damages caused by the *Phytoplasma* in the infected potato tubers and enhanced the quality, as well as the tuber yield [15,16]. The previous study illustrated that AsA reduces the severity of *Alternaria brassicicola* infection in *Arabidopsis* [17]. Moreover, they reported that *vtc1* and *vtc2* mutant *Arabidopsis* plants were highly sensitive to this pathogen due to a lower AsA level, which in turn causes increased cell damage. Accumulation of ascorbic acid provides a defense response to turnip mosaic virus (TMV) in resistant *Brassica rapa* [18]. The eggplant mottled dwarf virus affects ascorbic acid quality, plant development, and fruit ripening stages in *Solanum lycopersicon* [19]. Mohammed et al. noticed an inverse correlation between the rate of *P. infestans* infection and the antioxidant level in tomato (*Lycopersicon esculentum* Mill.) [20]. Previous studies demonstrated the better performance of *D-galacturonic acid reductase* (*GalUR*) transgenic plants under various abiotic stresses [21]. The aforementioned previous findings showed the positive effects of AsA in disease resistance against several plant pathogens. Hence, to determine their performance under biotic stress conditions, we subjected the control and transgenic potato plants to the *P. infestans* infection, which is the most common pathogen in potato. We observed a reduction in disease severity in the *GalUR* transgenic potato plants against the potato late blight disease. The molecular analysis of genes related to the pathogenesis resistance (*PR*) and hormone biosynthesis revealed their altered gene expression in response to the *P. infestans* attack. Furthermore, transgenic potato plants with enhanced hydrogen peroxide scavenging activity minimizes the foliar damage caused by the *P. infestans* infection.

## 2. Materials and Methods

### 2.1. Plant Material 

Transgenic *GalUR* potato (*Solanum tuberosum* L. cv. Taedong Valley) tubers over accumulating AsA (Ascorbate) were kindly provided [21,22]. The tubers were sprouted and grown to maturity in the glasshouse conditions. The single-node cuttings from the transgenic potato were cultivated in culture tubes (25 × 150 mm) on Murashige and Skoog (MS) basal medium [23] with 3% (w/v) sucrose. The subcultured shoots were kept in a plant growth chamber (22 ± 2 °C, light/dark cycle of 16 h/8 h). Two months-old in vitro grown *GalUR* transgenic and untransformed (UT) control plants (15 sets with three replicates) were transplanted into the soil and further maintained in the glasshouse, and were used for all experiments.

### 2.2. Phytophthora infestans Disease Resistance Studies 

*P. infestans* strain (KACC 40718) acquired from the Korean Agricultural Culture Collection (KACC), Rural Development Administration (RDA), South Korea was routinely subcultured in the rye-agar medium with sucrose (2%) and incubated at 18 ± 1 °C in the dark. The zoospores from 14 day-old cultures were isolated by flooding the culture plate with cold sterile distilled water followed by incubation at 4 °C for 2 h. The solution containing zoospores was removed from the Petri dish and kept on ice until used for potato leaf inoculation. Two months-old in vitro-grown *GalUR* transgenic and UT control potato plants (15 sets of *GalUR* transgenic and UT control plants with three replicates) were used for the challenging study. The fully developed leaves from the top third to fifth were detached and inoculated with 10 µL spots (1 × 10^5^ zoospores mL^−1^ concentration) of freshly isolated zoospores suspension on to the abaxial surface. Then the inoculated leaves were kept in water-saturated filter paper in the sterile Petri dishes. Parallelly, the well-developed leaves third from the top of the intact plants were inoculated with 10 µL of zoospore suspension. The inoculated plants were incubated at 18 ± 1 °C with a 16 h/8 h photoperiod with 95–98% relative humidity (RH) for one day and subsequently maintained at 70% RH. The development of disease and necrosis was examined carefully every day after pathogen inoculation. 

### 2.3. Histological Studies of Infected Leaves

Leaf discs from the infected leaves were stained with lactophenol–trypan blue staining, as described previously [24]. Stained specimens were examined under light microscopy for *P. infestans* infection. 

### 2.4. Evaluation of Disease Severity 

The scoring values for the disease severity in the *P. infestans* infected leaves of *GalUR* and UT control potato plants were given based on the previous study [25]. The infected plants were monitored after challenging with the pathogen. The disease severity index (DSI) scoring values (0–5) for the estimation of disease severity were assigned as follows: 0—no visible symptoms or lesions, 1—scarce visible lesions, 2—small lesions with 1% discoloration of leaves, 3—discoloration of leaves up to 10% with increased lesion size, 4—increased leaf discoloration (25%), 5—increased leaf discoloration (>25%), and severe damage. Each treatment had six replicates, and the experiment was repeated twice. The data are presented as means ± standard deviation of n = 6. The data were analyzed using the Kruskall–Wallis non-parametric test at *p* = 0.002.

### 2.5. Gene Expression Studies in Infected Samples

Total RNA was extracted from the *P. infestans* challenged leaf samples of UT control, as well as *GalUR* transgenic plants using Tri-reagent (Sigma Aldrich) followed by treatment with DNase I. First-strand cDNA synthesis, was carried out using 2 µg of total RNA, oligo dT, and Super Script-II Reverse Transcriptase (Invitrogen). Quantitative real-time polymerase chain reaction (qRT-PCR) analysis was performed using the SYBR PCR kit (Bio-Rad, Hercules) in the CFX 96 Touch Real-Time PCR Detection System (Bio-Rad, Hercules). The PCR reaction conditions are as follows; 95 °C for 10 s, then 38 cycles of 95 °C for 5 s, annealing temperature for 15 s, and 72 °C for 30 s. The primers used for the analysis of the transcripts variation are listed in Table 1. The gene expression variation for *PR1* (pathogenesis-related protein 1) and antimicrobial producing *PAL* (phenylalanine ammonia-lyase), antioxidative genes such as *APX* (ascorbate peroxidase), *GR* (glutathione reductase), *DHAR* (dehydroascorbate reductase), *CAT* (catalase), *SOD* (superoxide dismutase), *NCED1* (9-cis-epoxycarotenoid dioxygenase 1), and *NCED2* (both the *NCED1* and *NCED2* involved in the catalysis of ABA precursor zeaxanthin biosynthesis), *GA20OX1* (involved in the catalysis of GA biosynthesis) were analyzed. The relative gene expressions obtained by the comparative cycles of threshold (Ct) values of *GalUR* transgenic samples were normalized with the Ct values of the control (WT) plant samples, and the relative gene expression was calculated by using the 2^−∆∆Ct^ method [26]. The experiment was carried out separately in triplicates and was expressed as the mean ± standard deviation (SD). One-way ANOVA analysis followed by Duncan’s test was used to determine significant differences at *p* ≤ 0.05 significance level.

### 2.6. Estimation of H_2_O_2_ and MDA

The quantification of H_2_O_2_ content in UT control and *GalUR* transgenic plant leaf tissues challenged with *P. infestans* was carried out using a method, as described previously [27]. The MDA content in the UT control and transgenic leaf tissues were determined, as described earlier [28]. The experiments were carried out separately in triplicates and were expressed as the mean ± standard deviation (SD). One-way ANOVA analysis followed by the Duncan’s test was used to determine significant differences at *p* ≤ 0.05 significance level. 

### 2.7. Estimation of the Plant Hormones GA and ABA

The plant hormones, GA and the ABA were estimated using the protocol as published by Tang et al. [29]. All the experiments were conducted with waters alliance E-2695 Chromatographic station and direct ultraviolet–visible (UV) absorbance detection unit. The HPLC system consisted of C18 reverse-phase column (150 mm × 4.6 mm, 5 μm) with a methanol gradient in 0.6% acetic acid and UV detector set at 254 nm. Standard ABA and GA_3_ were purchased from Sigma (USA). Three plant sets from three groups were used for the phytohormone analysis. The leaf tissues (100 mg fresh mass) of UT control, *GalUR* transgenic, and *GalUR* transgenic plants challenged with *P. infestans* were ground in liquid nitrogen, homogenized, and then extracted overnight with 30 mL of 80% cold aqueous methanol (<0 °C) in darkness at 4 °C following the standard protocol. The stock solutions of the hormones were prepared at 1.0 mg/mL by dissolving accurately weighed amounts of each compound in methanol (MeOH) and stored at 4 °C. The experiments were carried out separately in triplicates and were expressed as the mean ± standard deviation (SD). One-way ANOVA analysis followed by Duncan’s test was used to determine significant differences at *p* ≤ 0.05 significance level.

### 2.8. Statistical Analysis

The experiments include real-time gene expression, HPLC, and biochemical estimations (H_2_O_2_ and MDA) which were done separately in triplicates and are expressed as the mean ± standard deviation (SD). One-way ANOVA analysis followed by Duncan’s test was used to determine significant differences at *p* ≤ 0.05 significance level. Disease severity index data were analyzed using the Kruskal–Wallis non-parametric test at *p* = 0.002. The statistical analyses were performed separately using the SPSS package program version 25 (SPSS, Chicago, IL, USA).

## 3. Results

### 3.1. GalUR Transgenics with Reduced Necrotic Damage to Fungal Pathogen Infection

**Figure 1:**
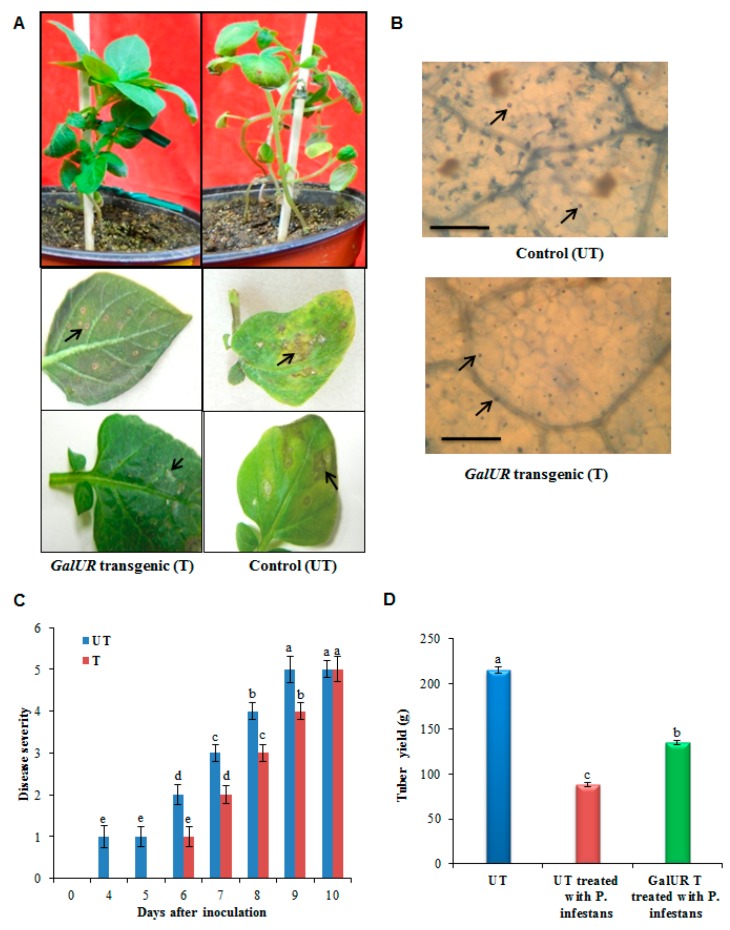
Pathogen infection in control and *GalUR potato* challenged with *P. infestans.* (**A**) *GalUR* transgenic potato plants showing restricted lesions and necrosis development (arrows) compared to control plants, (**B**) Microscopic appearances of *P. infestans* infected potato leaves stained with lactophenol-Trypan blue. The numbers of dead cells were seen in control plants while few dead cells were present in the *GalUR* potato transgenics after *P. infestans* infection. Bars = 200 µm, (**C**) Disease severity in potato plants. Disease range of damage caused by *P. infestans* after inoculation of leaves of potato plants. The disease severity index (DSI) scoring values (0–5) for the estimation of disease severity were assigned as follows: 0—no visible symptoms or lesions, 1 —scarce visible lesions, 2—small lesions with 1% discoloration of leaves, 3—discoloration of leaves up to 10% with increased lesion size, 4—increased leaf discoloration (25%), 5—increased leaf discoloration (>25%), and severe damage. Each bar represents a means ± standard deviation of n = 6. Different letters indicate a significant difference in disease severity according to a Kruskal–Wallis non-parametric test at *p* = 0.002, (**D**) Tubers yield in the untransformed (UT) control and *GalUR* transgenic potato challenged with *P. infestans* and UT control plants and the data are presented as means ± SD of three replicates. Different letters indicate a significant difference at *p* ≤ 0.05.

In the present investigation, both transgenic and the UT control plants showed small but visible lesions after four to five days of pathogen inoculation, indicating the successful pathogen infection (Figure 1A). Microscopic visualization revealed the pathogen-induced cell death in the infected leaf tissues. The occurrence of pathogen-induced cell death was significantly higher in the UT control potato leaves compared to the *GalUR* transgenic potato leaves (Figure 1B). The disease symptom also appeared quicker in UT control plants as compared to the transgenic plants, probably due to the high antioxidant activity observed in the *GalUR* plants. The lesion size progressively expanded until it became necrotic in the UT control plants, whereas, the infected leaves of thetransgenic plants exhibited reduced lesions and necrosis at the initial stages of pathogenesis (Figure 1A). This indicates that the increased antioxidant potential of *GalUR* expressing transgenic potato plants to the symptoms caused by *P. infestans*. Furthermore, the disease severity assessment showed that disease incidence started earlier in the control plants (UT) compared to *GalUR* transgenic plants. The progressive reduction of necrotic lesions was observed at earlier stages in the *GalUR* transgenic plants (Figure 1C). Both the UT control and transgenic plants could not diminish the occurrence of the disease; however, a delayed disease progression was observed in the case of the transgenic potato with enhanced L-AsA accumulation (Figure 1C). The tubers were also harvested from both the plants; however, the UT control potato suffered a huge loss of tubers than the transgenic potato (*P* ≤ 0.05). The untransformed control potato (cultivars Taedong Valley) yielded 215 grams of tubers under the control condition, while the diseased plant yielded 88.2 grams of tubers of smaller size. In contrast, the transgenic potato also suffered yield penalty, which yielded 135 grams tubers which were normal in size (Figure 1D).

### 3.2. Reduced Levels of H_2_O_2_ and MDA and Enhanced Antioxidant Gene Expression in GalUR-Transgenic Potato Plants

**Figure 2:**
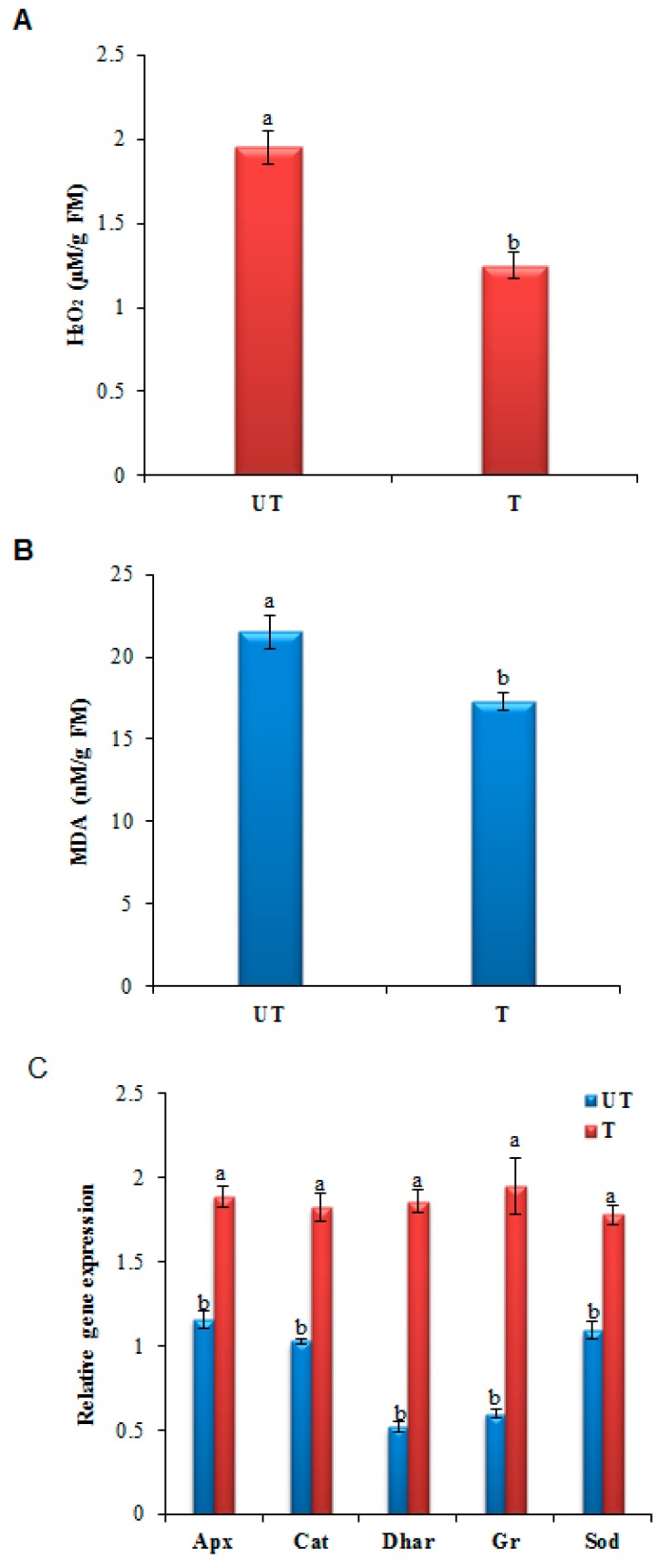
Histograms showing estimated (**A**) H_2_O_2,_ (**B**) MDA content in *P. infestans* infected *GalUR* transgenic (T) and untransformed control (UT) potato leaves, (**C**) genes encoding reactive oxygen species (ROS)-scavenging enzymes, ascorbate peroxidase (*APx*), catalase (*CAT*), dehydroascorbate reductase (*DHAR*), glutathione reductase (*GR*), and superoxide dismutase (*SOD*) in *P. infestans* infected *GalUR* transgenic (T) and untransformed control (UT) potato plants. Data are presented as means ± SD of three replicates. Different letters indicate a significant difference at *p* ≤ 0.05.

H_2_O_2_ is one of the major ROS molecules produced in plants in response to pathogen attack and its concentration was probably below the threshold in the *GalUR* transgenic potato lines (Figure 2A) due to its removal via the enhanced activity of the cellular antioxidants enzymes and AsA level, which probably protected the plant cells from further cellular damage. The level of MDA recognized as an indicator of lipid peroxidation. The MDA level was lower in *GalUR* transgenic plants than control plants (Figure 2B). The lower H_2_O_2_ and MDA content in transgenic plants compared to the UT control plants (*p* ≤ 0.05) indicated the enhanced ROS-scavenging activity via the maintenance of higher cellular AsA and the antioxidant enzymes, as evident in the gene expression analysis of the antioxidative genes such as *APX* (ascorbate peroxidase), *GR* (glutathione reductase), *DHAR* (dehydroascorbate reductase), *CAT*, and *SOD* (Figure 2C). In addition, the ROS scavenging activity is also associated with the reduced necrotic lesions observed in *GalUR* transgenics.

### 3.3. Altered Expression of PR, Phytohomone Genes, and Estimation of Phytohormones Using HPLC

**Figure 3:**
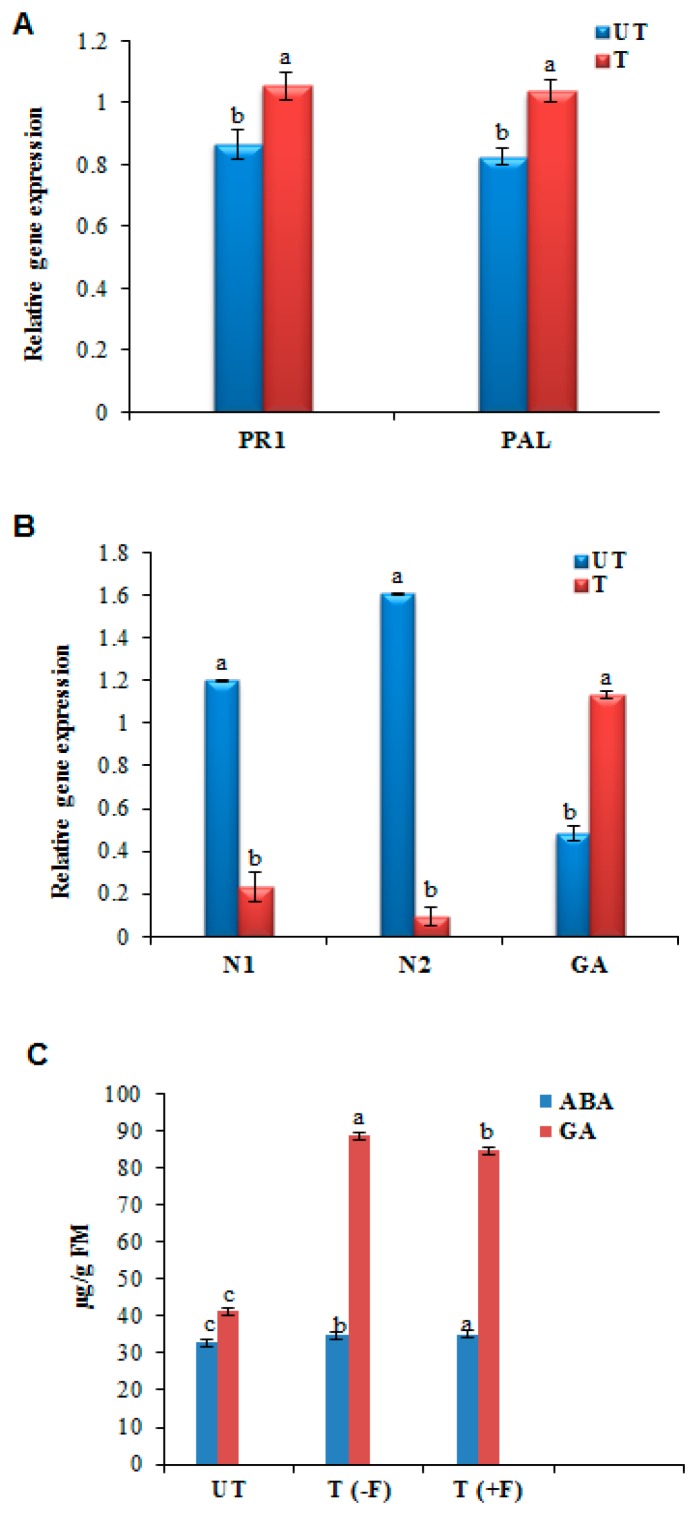
Quantitative Real-Time gene expression analysis of (**A**) Pathogenesis related (*PR*) genes, *PR-1* and *PAL* (phenylalanine ammonia-lyase), (**B**) Abscisic acid (ABA) biosynthesis pathway genes, *N1* (9-cis-epoxycarotenoid dioxygenase 1), *N2* (9-cis-epoxycarotenoid dioxygenase 2), and GA biosynthesis pathway gene (Gibberellic acid 20 oxidase), and (**C**) Estimation of phytohormones such as ABA and GA hormone in the in control (UT) and *GalUR* transgenic (T) transgenic potato. T (-F) denotes the transgenic plant without *P. infestans*, and T (+F) denotes the plant infected with the fungal strain. Data are presented as means ± SD of three replicates. Different letters indicate a significant difference at *p* ≤ 0.05.

Gene expression profiles of *PR1* and antimicrobial producing *PAL* (phenylalanine ammonia-lyase) gene was analyzed and the results indicated that the transcripts of *PR1* and *PAL* gene were slightly higher in the *GalUR* transgenics compared to UT control plants (*p* ≤ 0.05) (Figure 3A). Since it is known that AsA acts as a cofactor for several phytohormone biosyntheses and they play a certain role in plant immunity, we performed qRT-PCR analysis of genes that are intricate in the regulation of ABA and GA biosyntheses to monitor their transcript levels upon pathogen induction. *GA20OX1* expression was higher in the *GalUR* transgenic and lower in the control plants, whereas the reversible results were observed in the *NCED1, NCED2* expression (*p* ≤ 0.05) (Figure 3B). The chromatographic estimation also showed the presence of the ABA and the GA hormones in the UT control and the *GalUR* transgenic potato. However, the GA content was more in the case of the *GalUR* transgenic plants than the UT control, which was 2.3 times more in the *GalUR* transgenic plants (*p* ≤ 0.05) (Figure 3C). This may be due to the enhanced level of the AsA in the *GalUR* transgenic plant, which is a negative regulator of the ABA and a positive regulator of the GA, and hence promotes GA biosynthesis. The gene expression data also showed the same result. 

## 4. Discussion

In the current investigation, we planned to study the putative role of AsA in disease tolerance against *P. infestans* using transgenic potato plants overexpressing *GalUR* gene. These transgenic potato plants overproduce 1.6–2-fold AsA and exhibit tolerance to various abiotic stresses due to the enhanced ROS-scavenging activity [30]. AsA plays a vital role in detoxification of H_2_O_2_ and MDA, which is one of the major ROS molecules produced under pathogen attack in plants to induce hypersensitive response (HR). HR acts as a positive signal to transmit this locally induced resistance at the systemic level. However, the higher H_2_O_2_ and MDA concentration should be detoxified by antioxidative systems before it acts as a potent toxic to plants. Here, we report the *GalUR* transgenic plants with reduced H_2_O_2_ and MDA content due to the efficient detoxification by L-ascorbate. 

The efficient ROS-scavenging activity of *GalUR* transgenics was evident from the upregulated transcripts of various antioxidative enzymes such as *SOD, GR, DHAR, APX,* and *CAT* compared to control plants (*p* ≤ 0.05) indicating the importance of antioxidative enzymes and their promising role in the detoxification of ROS (Figure 3C). Sarowar et al. revealed that the tobacco plants overexpressing *Capsicum annuum* ascorbate peroxidase-like-1 gene (*CAPOA1*) showed enhanced ROS-scavenging activity due to its two-fold higher peroxidase transcript level and provided increased resistance to *Phytophthora nicotianae* [31]. Similarly, Zhu et al. suggested that the induction of antioxidant genes, such as *DHAR* and *GR* genes were involved in the alleviation of the RNA virus induced disease symptoms [32]. Consistent with our study, we observed the high transcript abundance of peroxidase (*APX*), *DHAR,* and *GR* genes in *GalUR* transgenics, which directly corresponds to the reduction in disease symptoms. These *GalUR* transgenics showed reduced necrotic spots compared to the control leaf samples due to the enhanced active oxygen scavenging system. Microscopic visualization analysis showed that pathogen-induced cell death was higher in control plants than *GalUR* transgenic leaves. Moreover, the lesion formation and its proliferation were delayed in the *GalUR* transgenic plants. These results clearly indicate the potential scavenging activity of AsA on pathogen-induced excessive ROS molecules. The present observation also overlaps with the previous study, which stated that the exogenous application of AsA reduces the severity of blight diseases and the oomycetes spore count [33]. Another recent study also demonstrated that the AsA treatment at a higher concentration in the initial stage protects plants through effective alleviation of disease symptoms, as well as the inhibition of viral replication [34]. However, in the current investigation, *GalUR* transgenic plants showed reduced disease severity at the initial stages but did not control the disease. 

It has been suggested that *PR* genes such as *PR1*, *PR5,* and *PAL* accumulate after pathogen attack and play a positive role in conferring resistance against *P. infestans* [35]. However, in the present study, a slightly higher expression of *PR1* and *PAL* gene transcripts was observed in the non-transgenic plants than the *GalUR* transgenics perhaps due to the higher H_2_O_2_ content in the control potato plants (*P*≤0.05). AsA acts as a cofactor for the enzymes involved in ABA and GA biosynthesis pathway, it can influence these hormone levels and their signaling pathways. It was reported that the high AsA level downregulates ABA biosynthesis and promotes GA biosynthesis [36]. In accordance with these results, upregulation of *GA20Ox1* transcripts and downregulation of *NCED1* and *NCED2* transcripts were found in the *GalUR* potato transgenics (*P*≤0.05). The GA and ABA act as a positive signal for the biotic stress resistance, which was proved in various crops [37,38]. Mohr and Cahill suggested that the pretreatment of the ABA significantly decreased the production of salicylic acid and the lignification’s process in *Arabidopsis* infected with the *Pseudomonas syringae* pv. *tomato* [39]. However, emerging evidence suggests that GA signaling components play major roles in plant disease resistance and susceptibility. Recently, it has been found that *Arabidopsis* DELLA (aspartic acid–glutamic acid–leucine–leucine–alanine) proteins, which act as negative regulators of GA signaling, control plant immune responses by modulating salicylic acid (SA) and jasmonic acid (JA) dependent defense responses [40]. Moreover, it has been shown that DELLA proteins promote the expression of genes encoding ROS detoxification enzymes, thereby regulating the levels of ROS after biotic or abiotic stress [41]. In consistence with this, the transgenic potato showed lesser necrotic lesions due to the ROS removal via maintenance of the higher L-ascorbate, higher antioxidant enzymes, and enhanced GA level, which not only removed the H_2_O_2_ or other ROS, but the disease resistance and susceptibility against the fungus due to GA signaling pathways. It has also been demonstrated that *gid1* mutant of rice, defective in GA receptors, accumulates higher GA levels, and shows enhanced resistance to the blast fungus *Magnaporthe* *grisea* compared to wild type plants [42]. Consistent with these reports, we observed the higher GA and lower ABA levels in the *GalUR* transgenic plants might also be the responsible factor for the delayed disease progression in *GalUR* transgenic plants. The strong ROS detoxification mechanism found in *GalUR* transgenics reduces the cell death and necrotic symptoms, which in turn reduce the severity of the disease.

## 5. Conclusions 

*GalUR* transgenic potato plants overproducing AsA with potent antioxidant capacity showed decreased necrotic symptoms and reduced disease lesions in the initial stages induced by *P. infestans* challenge. These plants exhibited enhanced active oxygen scavenging activity via the increased accumulation of antioxidative enzymes that lead to the efficient detoxification of H_2_O_2_, which could otherwise cause severe necrotic death of disease-infected leaves. These plants showed altered hormone biosynthesis, such as the upregulation of the GA and downregulation of ABA. Further, the defense-related *PR1* and *PAL* gene expression was slightly lower in *GalUR* transgenic plants as compared to control plants. Our findings suggest that the decreased necrotic cell death associated with the reduction in foliar tissue damage was induced through the efficient antioxidant system of the *GalUR* transgenic potato plants, although it does not essentially control the pathogen growth and disease. Further characterization of these plants is warranted to understand the molecular basis of biotic stress tolerance in plants that accumulate higher levels of AsA.

## Figures and Tables

**Table 1:** List of primers used in the study.

NCBI Accession Number	Primer Name	Sequence (5′–3′)
X55749	*Actin*	F: CTGGTGGTGCAACAACCTTA
		R: GAATGGAAGCAGCTGGAATC
AB041343	*APx*	F: ACCAATTGGCTGGTGTTGTT
		R: TCACAAACACGTCCCTCAAA
AY442179	*CAT*	F: TGCCCTTCTATTGTGGTTCC
		R: GATGAGCACACTTTGGAGGA
AF354748	*SOD*	F: GTTTGTGGCACCATCCTCTT
		R: GTGGTCCTGTTGACATGCAG
X76533	*GR*	F: GGATCCTCATACGGTGGATG
		R: TTAGGCTTCGTTGGCAAATC
DQ512964	*DHAR*	F: AGGTGAACCCAGAAGGGAAA
		R: TATTTTCGAGCCCACAGAGG
AJ250136	*PR1*	F: GCATCCCGAGCACAAAATTA
		R: GAAATCACCACTTCCCTTGG
X63103	*PAL1*	F: TTGCACAAGTTGCATCCATT
		R: CACCAGCTCTTGCACTTTCA
AJ291453	*GA20OX1*	F: CAAGATTGTGTTGGCGGACT
		R: ACTGCTCTGTGCAGGCAACT
AY662342	*StNCED1*	F: GGAAATCAACAAGAAAAGCCA
		R: ATATTTGTTGTCACCATAAATGAA
AY662343	*StNCED2*	F: GGGACTTTCATTAGCTCAAAGGACTTGC
		R: GCGATGTAAATTTGAATTACTATTATTCGCTCA
